# Task-oriented exercises improve disability of working patients with surgically-treated proximal humeral fractures. A randomized controlled trial with one-year follow-up

**DOI:** 10.1186/s12891-021-04140-9

**Published:** 2021-03-20

**Authors:** Marco Monticone, Igor Portoghese, Daniele Cazzaniga, Valentina Liquori, Giuseppe Marongiu, Antonio Capone, Marcello Campagna, Giovanni Zatti

**Affiliations:** 1 Department Medical Sciences and Public Health, University of Cagliari, Cittadella Universitaria, Strada Statale, 554 - Monserrato, Cagliari, Italy; 2 Neurorehabilitation Unit, Department Neuroscience and Rehabilitation, G. Brotzu Hospital, Cagliari, Italy; 3 Istituti Clinici Scientifici Maugeri IRCCS, Lissone, Italy; 4 Orthopaedic and Trauma Clinic, Department of Surgical Sciences, University of Cagliari, Cagliari, Italy; 5 Bicocca University of Milan, Milan, Italy; 6 Orthopaedics Unit, San Gerardo Hospital, Brianza, Monza, Italy

**Keywords:** Proximal humeral fractures, Surgery, Rehabilitation, Task-oriented exercises

## Abstract

**Background:**

General physiotherapy is a common means of rehabilitation after surgery for proximal humeral fracture (PHF). Better-targeted exercises seem worthy of investigation and the aim of this study was to assess the efficacy of a rehabilitation program including task-oriented exercises in improving disability, pain, and quality of life in patients after a PHF.

**Methods:**

By means of a randomized controlled trial with one-year follow-up, 70 working patients (mean age of 49 ± 11 years; 41 females), who were selected for open reduction and internal fixation with plates caused by PHF, were randomized to be included in an experimental (*n* = 35) or control group (n = 35). There was a permuted-block randomization plan, and a list of program codes was previously created; subsequently, an automatic assignment system was used to conceal the allocation. The first group underwent a supervised rehabilitation program of task-oriented exercises based on patients’ specific job activities, and occupational therapy. The second group underwent general physiotherapy, including supervised mobility, strengthening and stretching exercises. Both groups individually followed programs of 60-min session three times per week for 12 weeks in the outpatient setting. The Disability Arm Shoulder Hand questionnaire (DASH; scores range from 0 to 100; primary outcome), a Pain intensity Numerical Rating Scale (scores range 0 to 10; secondary outcomes), and the Short-Form Health Survey (scores range from 0 to 100; secondary outcomes) assessed the interventions. Participants were evaluated before surgery, before and after rehabilitation (primary endpoint), and at the one-year follow-up (secondary endpoint). A linear mixed model analysis for repeated measures was carried out for each outcome measure (*p* < 0.05).

**Results:**

Time, group and time by group showed significant effects for all outcome measures in favour of the experimental group. The DASH and the DASH work achieved clinically important between-group differences of 16.0 points (95% confidence interval [C.I.] 7.3 to 24.7) and 19.7 (95% C.I. 9.0 to 30.5) at follow-up, respectively. The NRS achieved a between-group difference of 2.9 (95% C.I. 1.0 to 3.9) at follow-up. As for SF-36, there were between-group differences ranging from 17.9 to 37.0 at follow-up.

**Conclusions:**

A rehabilitation program based on task-oriented exercises was useful in improving disability, pain, and quality of life in working patients after PHFs. Improvements lasted for at least 12 months.

**Trial registration:**

On 16/12/2019, the trial was retrospectively registered in the ISRCTN registry with the ID number 17996552.

## Background

Upper limb fractures are continuously increasing in industrialized western countries: about 370,000 visits to the emergency departments are expected to occur every year in the United States [1]. The most common site for fracture is proximal humerus accounting for 50% of them, with higher rates in females (78 visits with humeral fractures/100,000 people) than in males (36 visits with humeral fractures/100,000 people), and with a larger number in the 45–64 years age group [1]. Proximal humeral fractures (PHFs) induce pain and limitations in activities of daily living (ADL), and reduce quality of life (QoL) [1, 2]. In addition, PHFs produce relevant direct costs including medical costs: the hospitalization cost is the most important factor in total healthcare cost of PHFs, being 55% of the total healthcare costs [3]. Despite exact estimates of indirect costs not being available, presumably long-term loss of earnings, vocational rehabilitation expenditures, pensions and wage-replacement costs, production slowdowns, accident investigations, and finally, the recruiting and training of workers to replace those injured are also expected [4].

PHFs advocate frequent demand for subsequent surgical and rehabilitation treatments [5]. When dealing with adult working patients it is common to firstly introduce joint-saving procedures, including minimally invasive reduction and intramedullary fixation, open reduction and internal plates fixation [6, 7]. Subsequently, non-operative treatments are recommended, usually based on immobilization and then on rehabilitation [6, 7]. An efficient return-to-work time is also recommended to decrease individual and company costs [3].

General physiotherapy is a common means of rehabilitation after surgery for PHFs, and mainly includes segmentary exercises of mobility of the shoulder and upper limb, strengthening of humeral and upper limb muscles, stretching of shoulder girdle and upper limb muscles, and postural control of upper limb [7–10]. However, its clinical impact relies on the recovery of general motor characteristics and not of specific daily living and job activities, useful when working populations are addressed [7]. Hence, better-targeted exercises, such as those task-oriented, whose aim is the recovery of specific movements performed during job activities as well as early independence in ADL, seem worthy of investigation in this context. Evidence is also required when defining other characteristics of these exercises, such as intensity, frequency and duration of programs as well as their long-term effects [7].

Hence, we carried out a randomized controlled study with the objective of assessing the efficacy of a multimodal rehabilitation program that incorporates task-oriented exercises and occupational therapy compared to general physiotherapy in improving disability (primary outcome), pain and quality of life (secondary outcomes) of working patients who had PHF surgically-treated with locking plate fixation. The hypothesis was that a 12-week rehabilitation program of functional exercises would produce clinically significant improvements in the primary outcome versus general physiotherapy at the end of treatment (primary endpoint), and that these effects would have lasted up to at least one year (secondary endpoint).

## Methods

### Design, randomization and blinding

A secondary care rehabilitation hospital was the setting of our randomized, parallel-group superiority-controlled trial, in accordance with the CONSORT recommendations [11]. The staff involved had documented expertise in upper limb rehabilitation (over 10 years of practice) and attended refresher courses which combined theory with practical treatment skills on post-surgical management of musculoskeletal disorders annually.

Once the patients gave their consent, the principal investigator informed the biostatistician, who randomized them to one of the interventions using a permuted-block randomization plan. The list of program codes was previously created by the same researcher and stored in Matlab; the sequence was not accessible to others involved in the study. Subsequently, an automatic assignment system, also created in Matlab, was used to conceal the allocation.

The principal investigator, who acquired and evaluated the data was blinded to the treatment allocation. The remaining health-care personnel, the biostatistician and the participants were aware of the treatment and, therefore, were not blinded.

Our study was approved by the Local Ethical Committee “Salvatore Maugeri Foundation”, Scientific Institute of Lissone (MB, Italy; number: 4; date of approval: 04/05/12) and was carried out observing the fundamental ethical and humane principles of research. The trial was retrospectively registered in the ISRCTN registry with the ID number 17996552 (16/12/2019). A protocol was presented to the Local Ethical Committee before the study began and no changes occurred over the course of the study.

### Patients

Patients included were those undergoing surgical treatment of open reduction and internal fixation with a locking plate as a result of displaced and unstable PHFs classified as Arbeitsgemeinschaft Osteosynthese (AO) Foundation for the Study of Internal Fixation type 11 [12], those who had a job, a good understanding of Italian and an age between 20 and 65. The exclusion criteria were as follows: cognitive impairment (Mini-Mental State Examination Score of < 24); unstable cardiovascular and pulmonary diseases; systemic or neuromuscular diseases (including rheumatic diseases), ruled out by case histories and imaging. Patients with isolated tuberculum majus fracture (AO 11, A1), those with fractures involving the glenoid cavity, double fractures or injury of the plexus and the axillary nerve, and those with workers’ compensation were also not included.

The study’s participants were consecutively included in the study between June 2012 and June 2016.

They were pre-operatively assessed by an orthopaedic surgeon and a physiatrist at the Orthopaedics Unit (San Gerardo Hospital, Monza, Italy), and all those who fulfilled the entry criteria received additional information and were asked to agree to comply with whichever rehabilitation option conducted at the Rehabilitation Unit (Scientific Institute of Lissone, Salvatore Maugeri Foundation, Lissone, Italy) they were randomly assigned to, and finally, to attend the follow-up examination. The patients were blinded to the study’s hypothesis to partially reduce expectation bias and crossover problems. They were told that the trial’s objective was to compare two common approaches to PHFs rehabilitation, whose efficacy had not yet been established. Conforming patients were required to sign an informed consent form, and subsequently, their demographic data, symptoms and medical history were taken down.

### Interventional programs

The interventional programs involved two physiatrists, an occupational therapist, and two equally-experienced physiotherapists who were individually responsible for the intervention in the two different programs. The exercise program was designed on physical examination at baseline of postural, muscular, and articular performances .

#### Experimental group

After early mobilization (by the end of the first week after surgery), basic exercises were introduced to improve glenohumeral mobility and upper limb muscle awareness; the patients specifically learned techniques for the muscles mainly involved, progressively increasing resistance, speed, power and complexity of movement patterns. Further, the physiotherapist introduced task-oriented exercises based on patients’ specific job activities the patients accurately described. The exercises included: moving objects of different shapes and sizes over different directions, picking up objects on a table at different positions, composing complex objects by merging components placed on a table, catching objects thrown at different heights and velocities. Those physical movements aimed at gradually improving mobility and strength, recovering segmental stretching, and improving neuromotor control of the upper limb as a whole. Additional exercises were introduced with the aim of recovering dexterity, balance, and other functional demands of ADL. They included: moving from a couch to the sitting position and from a chair to a standing position, walking and turning at the preferred speed, ascending/descending stairs, and climbing obstacles. In addition, the occupational therapist gave patients sling care information as well as ergonomic principles based on the working activities actually performed before the injury.

#### Control group

After early mobilization (by the end of the first week after surgery), this program incorporated segmentary exercises for humeral mobilisation (including passive mobilization to improve glenohumeral range of motion), strengthening (involving humeral and upper limb muscles), segmentary muscle stretching (including upper limb and back muscles), and postural control (involving exercises to develop motor control of upper limb and the cervico-thoracic spine). Also, in this case sling care information was delivered.

The interventional programs were performed individually and lasted twelve weeks: both groups were involved in three, individual 1- h sessions a week of physical training (medium intensity), attending a total of 36 meetings; moreover, the experimental group met with the occupational therapist once a week for a 60-min session. At the end, participants were asked to actively perform the learnt exercises at home. During each session and at the end of the programs, a fidelity check, based on a treatment manual for the administration of exercise training, was carried out. This was necessary in order to control for variability in the treatment administration throughout the entire trial.

Full description of both interventions is available in the Appendix.

The patients, once accepted for the program, did not receive any other treatments (e.g. physical modalities, shoulder injections or nerve blocks); they were not allowed to take drugs such as steroids, opioids, anticonvulsants and antidepressant analgesics. However, they were allowed to take mild analgesics (e.g. acetaminophen) and non-steroidal anti-inflammatory drugs. General Practitioners were also asked to avoid prescribing any other treatments while the participants were involved in the programs. Spouses, partners or parents were invited to encourage patients’ compliance throughout the study and to promptly notify staff of any complication or difficulty experienced by the patient, in order to enhance treatment adhesion and reduce drop-outs. In order to enhance compliance, the participants also filled out a diary, which was checked by the physiotherapists every week, after each training session.

### Outcome measures

Disability (considered as the primary outcome) was evaluated via the validated Italian version of the self-administered 30-item Disability Arm Shoulder Hand questionnaire (DASH) [13, 14], which allows a comprehensive evaluation of upper limb problems; in the aforementioned questionnaire the total score ranges from 0 (no disability) to 100 (maximum disability). The work-related troubles subscale (4-item tool, DASH work; secondary outcome) was also completed; the total score varies again from 0 (no limitation on the patient’s ability to work) to 100 (maximum limitation).

Pain intensity (secondary outcome) was collected with the 11-point Numerical Rating Scale (NRS) ranging from 0 (no pain at all) to 10 (the worst imaginable pain) [15]. Patients were asked to evaluate their current pain.

QoL (secondary outcome) was evaluated with the use of the Italian version of the self-administered Short-Form Health Survey (SF-36). This measure is made up of eight different domains and scores were calculated on the basis of the User’s Manual for the Italian version, for which 0 represents the worst perceived QoL, and 100 the best perceived QoL [16, 17].

The scales were completed in 4 different times: before surgery and rehabilitation programs, 12 weeks later (post-training; primary endpoint), and 12 months (12 M follow-up) after the end of rehabilitation (secondary endpoint).

During the treatment period, the questionnaires were administered by secretaries who checked them and returned any uncompleted part to the patients for completion. At follow-up, the patients returned to the Rehabilitation Unit or were contacted by phone by the same secretaries in order to complete the questionnaires. The secretaries administrating the questionnaires were blinded to treatment allocation.

At the end of the treatment, patients rated the efficacy of the treatment using the Global Perceived Effect scale (GPE; secondary outcome), by means of a 7-point Likert scale (1 = helped a lot, 2 = helped, 3 = helped only a little, 4 = did not help, 5 = made things a bit worse, 6 = made things worse, 7 = made things a lot worse) [18].

Finally, using a specific form, we encouraged patients to notify us of any serious symptoms they experienced.

We analyzed the distribution of white- and blue- collar in the two groups, the DASH work values considering the type of work (blue- vs. white- collar) and whether the fracture was a work-related injury in order to avoid potential bias due to job-related biomechanical overload of the upper limb on returning to work. Specifically, we included high-skilled jobs (i.e. professional, administrative and managerial, clerical and sales) in the white-collar group, whereas low-skilled workers (transport and production workers and labourers) were in the blue-collar group.

### Statistics

We computed the sample size using the Italian version of the DASH, considering a between-group difference of 8 points as clinically important [6]. Sixty patients were required to guarantee 80% statistical power and 5% type I error, considering a standard deviation of 16 points [19]. To control for a drop-out of 10%, we enrolled 70 patients.

Linear mixed models for repeated measures (significance level of 5%) were analysed of each of the outcome measures. In this respect, group and time were entered as fixed effects, the patients as a random effect and the outcome measure as a dependent variable. Furthermore, we considered the time by group interaction term in our analyses. The linear mixed model was chosen in order to better deal with missing data since an intention-to-treat analysis was performed [20, 21]. Between-group differences at the end of the intervention and at follow-up were also investigated for both disability and other measures.

As GPE is a categorical measure, we analyzed it by using the Mann–Whitney U test. In order to evaluate the independence between nominal variables, we used Pearson’s chi square test at baseline.

The data were analyzed using SPSS software, version 26.

## Results

### Recruitment and baseline data

The study flowchart is reported in Fig. 1. No problems of crossover were shown as no participant requested to change groups. Baseline demographic, clinical characteristics and patient-rated outcomes are reported in Table 1 and Table 2.

**Figure:**
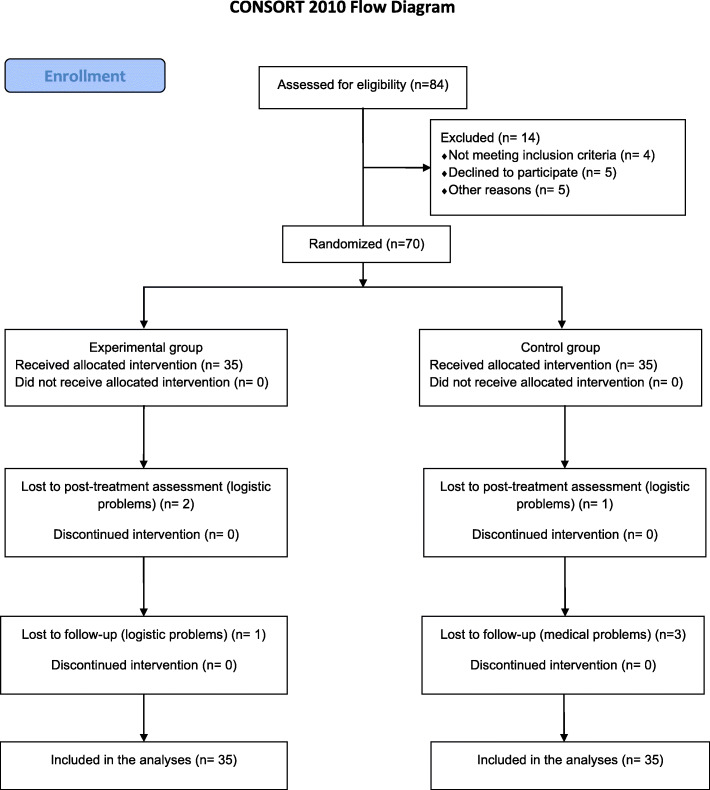
Fig. 1 Participants’ CONSORT flow chart

**Table 1:** Baseline demographic and clinical characteristics (*n* = 70)

		Experimental group (***n*** = 35)		Control group (***n*** = 35)	
**Age (Mean; Standard Deviation)**		50.57 (11.39)		47.71 (10.95)	
**Sex**	Male	13		16	
	Female	22		19	
**Employment**	Self-employed	15		13	
	Employed	20		22	
**Education level**	Primary school	3		3	
	Middle school	16		17	
	High school	10		11	
	University	6		4	
**Physical activity**	Yes	9		13	
	No	26		22	
**Arm injured**	Left	17		16	
	Right	18		19	
**Dominant arm**	Left	16		15	
	Right	19		20	
**Type of drug used**	Analgesics	7		6	
	NSAIDs	15		14	
	Muscle relaxant	13		15	
**Smokers**	Yes	16		24	
	No	19		11	
**Married**	Yes	26		28	
	No	9		7	
		**M (SD)**	**95% CI**	**M (SD)**	**95% CI**
**DASH (0–100)**		85.09 (7.65)	82.46–87.72	86.43 (7.44)	83.87–88.99
**DASH – WORK (0–100)**		93.04 (7.23)	90.55–95.52	91.07 (6.48)	88.84–93.30
**NRS (0–10)**		7.46 (1.48)	6.95–7.97	7.71 (1.54)	7.18–8.24
**SF-36 Physical function (0–100)**		33.29 (21.11)	26.04–40.54	27.29 (27.29)	17.91–36.66
**SF-36 Physical role (0–100)**		22.14 (34.18)	10.40–33,88	22.14 (32.52)	10.97–33.32
**SF-36 Bodily pain (0–100)**		22.37 (13.22)	17.83–26.91	22.57 (17.18)	16.67–28.47
**SF-36 General health (0–100)**		34.43 (19.94)	29.30–39.56	32.71 (13.95)	27.92–37.51
**SF-36 Vitality (0–100)**		30.43 (18.96)	23.92–36.94	29.57 (17.12)	23.69–35.45
**SF-36 Social function (0–100)**		45.36 (12.89)	40.93–49.79	45.71 (10.91)	41.97–49.46
**SF-36 Emotional role (0–100)**		15.24 (21.91)	7.71–22.76	13.33 (20.13)	6.42–20.25
**SF-36 Mental health (0–100)**		37.14 (13.94)	32.35–41.93	35.66 (12.70)	31.29–40.02

Note: *DASH* Disability Arm Shoulder Hand questionnaire; *NRS* Numerical Rating Scale; *NSAIDs* Non-steroidal anti-inflammatory drugs;

*SF-36* Short-Form Health Survey.; *M* Mean; *SD* Standard Deviation

**Table 2:** Patient-rated outcomes before rehabilitation (*n* = 70)

Measures	Before rehabilitation			
	Experimental group (*n* = 35)		Control group (*n* = 35)	
	M (sd)	95% CI	M (sd)	95% CI
DASH (0–100)	70.57 (15.72)	65.17–75.98	71.10 (16.33)	65.49–76.71
DASH – WORK (0–100)	78.75 (23.00)	70.85–86.65	78.04 (23.99)	69.80–86.28
NRS (0–10)	6.31 (2.46)	5.47–7.16	6.43 (2.44)	5.59–7.27
SF-36 Physical function (0–100)	32.43 (19.26)	25.81–39.05	30.57 (21.58)	23.16–37.99
SF-36 Physical role (0–100)	27.14 (30.54)	16.65–37.63	29.29 (27.44)	19.86–38.71
SF-36 Bodily pain (0–100)	37.37 (19.59)	30.64–44.10	35.37 (18.84)	28.90–41.84
SF-36 General health (0–100)	42.57 (14.06)	37.74–47.40	45.14 (10.47)	41.55–48.74
SF-36 Vitality (0–100)	47.29 (20.09)	40.39–54.19	51.71 (17.53)	45.69–57.74
SF-36 Social function (0–100)	51.79 (13.26)	47.23–56.34	47.14 (10.09)	43.68–50.61
SF-36 Emotional role (0–100)	28.57 (26.99)	19.30–37.84	26.67 (27.77)	17.13–36.21
SF-36 Mental health (0–100)	46.74 (16.88)	40.94–52.54	42.74 (14.32)	37.82–47.66

Note: *DASH* Disability Arm Shoulder Hand questionnaire; *NRS* Numerical Rating Scale; *SF-36* Short-Form Health Survey; *M* Mean; *sd* Standard Deviation

### Demographics and preoperative clinical characteristics

The sample consisted of 70 patients with a mean age of 49 ± 11 years (range: 23–67 years). 58,6% (*n* = 41) were females. 60% (*n* = 42) of the patients worked as employees and 47.1% (*n* = 33) had a middle school educational level.

Concerning the preoperative clinical characteristics, 55,7% (*n* = 39) of the patients were right-arm dominants and the injury occurred to the dominant arm in 56% (*n* = 22) of them. Whereas for left-arm dominants (44,3%, *n* = 31) the injury occurred to the dominant arm in 51,6% (*n* = 22) of them. Blue collars represented 53% (no. 37) of the sample, while white collars 47% (no. 33). Blue and white collars were equally distributed in the experimental and control group: 18 blue collars and 17 white collars, and 19 blue collars and 16 white collars, respectively.

Our sample showed mean DASH score of 85.76 (SD = 7.52), DASH-WORK = 92.05 (SD = 6.89), NRS = 7.59 (SD = 1.51), SF-36-physical functioning = 30.29 (SD = 24.40), SF-36-physical role = 22.14 (SD = 33.12), SF-36-bodily pain = 22.47 (SD = 15.22), SF-36-general health = 33.57 (SD = 14.37), SF-36-vitality = 30.00 (SD = 17.94), SF-36-social functioning = 45.54 (SD = 11.86), SF-36-emotional role = 14.29 (SD = 20.91), and SF-36-mental health = 36.40 (SD = 13.26).

### Outcome measures

The analysis showed a significant effect of time, group, and time by group interaction for all outcome measures (Tables 3 and 4). Furthermore, since no differences appeared from demographics, the analyses on intervention effects were unadjusted.

**Table 3:** Patient-rated outcomes at individual time points

Measures	Before rehabilitation				After 12 weeks				Follow-up 12 months			
	Experimental group (*n* = 35)		Control group (*n* = 35)		Experimental group (*n* = 35)		Control group (*n* = 35)		Experimental group (*n* = 35)		Control group (*n* = 35)	
	M (sd)	95% CI	M (sd)	95% CI	M (sd)	95% CI	M (sd)	95% CI	M (sd)	95% CI	M (sd)	95% CI
Disability Arm Shoulder Hand questionnaire (0–100)	70.57 (15.72)	65.17–75.98	71.10 (16.33)	65.49–76.71	28.65 (21.16)	21.15–36.15	41.78 (23.29)	33.65–49.91	20.06 (22.10)	12.09–28.03	36.02 (22,42)	27.80–44.24
Disability Arm Shoulder Hand questionnaire – WORK (0–100)	78.75 (23.00)	70.85–86.65	78.04 (23.99)	69.80–86.28	50.38 (26.24)	41.08–59.68	68.57 (24.93)	59.87–77.26	36.91 (27.10)	27.14–46.68	56.65 (26,37)	46.98–66.32
Numerical Rating Scale (0–10)	6.31 (2.46)	5.47–7.16	6.43 (2.44)	5.59–7.27	3.58 (1.90)	2.90–4.25	6.32 (2.40)	5.49–7.16	2.44 (1.90)	1.75–3.12	5.35 (2,30)	4.51–6.20
SF-36 Physical function (0–100)	32.43 (19.26)	25.81–39.05	30.57 (21.58)	23.16 37.99	71.67 (11.90)	67.45–75.89	47.35 (10.96)	43.53–51.18	79.84 (12.21)	75.44–84.25	58.06 (15,95)	52.21–63.92
SF-36 Physical role (0–100)	27.14 (30.54)	16.65–37.63	29.29 (27.44)	19.86–38.71	70.45 (19.22)	63.64–77.27	55.88 (16.35)	50.18–61.59	80.47 (13.82)	75.49–85.45	58.06 (19,78)	50.81–65.32
SF-36 Bodily pain (0–100)	37.37 (19.59)	30.64–44.10	35.37 (18.84)	28.90–41.84	48.76 (16.55)	42.89–54.63	29.97 (13.70)	25.19–34.75	56.88 (13.67)	51.95–61.80	34.71 (13,42)	29.79–39.63
SF-36 General health (0–100)	42.57 (14.06)	37.74–47.40	45.14 (10.47)	41.55–48.74	56.82 (9.42)	53.48–60.16	45.00 (11.48)	40.99–49.01	64.38 (11.76)	60.13–68.62	45.48 (12,74)	40.81–50.16
SF-36 Vitality (0–100)	47.29 (20.09)	40.39–54.19	51.71 (17.53)	45.69–57.74	66.82 (15.45)	61.34–72.30	52.06 (13.21)	47.45–56.67	78.75 (14.20)	73.63–83.87	52.58 (13,22)	47.73–57.43
SF-36 Social function (0–100)	51.79 (13.26)	47.23–56.34	47.14 (10.09)	43.68–50.61	69.32 (23.40)	61.02–77.62	58.46 (20.81)	51.20–65.72	80.08 (13.42)	75.24–84.92	58.47 (15,61)	52.74–64.19
SF-36 Emotional role (0–100)	28.57 (26.99)	19.30–37.84	26.67 (27.77)	17.13–36.21	73.74 (24.66)	64.99–82.48	39.22 (25.25)	30.40–48.08	84.38 (22.38)	76.31–92.44	47.31 (24,00)	38.51–56.11
SF-36 Mental health (0–100)	46.74 (16.88)	40.94–52.54	42.74 (14.32)	37.82–47.66	75.52 (12.02)	71.25–79.78	57.53 (14.61)	52.43–62.63	76.13 (15.69)	70.47–81.78	58.19 (14,38)	52.92–63.47

Note: *M* Mean; *sd* Standard Deviation; *DASH* Disability Arm Shoulder Hand questionnaire; *NRS* Numerical Rating Scale; *SF-36* Short Form Health Survey; group 1 = Experimental group (*n* = 35); group 2 = Control group (*n* = 35)

**Table 4:** Between-group differences from pre-rehabilitation to primary and secondary endpoints

		Mean difference pre Rehab			Mean difference after 12 weeks			Mean difference at follow up 12 months			F (***P***-value) time effect		F (***P***-value) group effect		F (***P***-value) interaction effect	
		M	95% C.I.		M	95% C.I.		M	95% C.I.		F	p	F	p	F	p
DASH	1 vs 2	−0.53	[−7.73; 8.78]	t(270) = − 0.318	−13.13	[4.69; 21.57]	t(270) = − 3.050	− 15.96	[7.26; 24.66]	t(270) = − 3.596	167.11	< 0.001	13.00	< 0.001	3.41	< 0.05
				*p* = 0.900			*p* < 0.01			*p* < 0.001						
DASH WORK	1 vs 2	0.71	[−10.87; 9.44]	t(270) = 0.360	− 18.19	[7.80; 28.57]	t(270) = −3.650	−19.74	[9.03; 30.45]	t(270) = − 3.712	220.34	< 0.001	12.51	< 0.001	3.46	< 0.05
				*p* = 0.835			*p* < 0.01			*p* < 0.001						
NRS	1 vs 2	−0.11	[−0.85; 1.08]	t(270) = −0.233	−2.75	[1.76; 3.73]	t(270) = −5.471	−2.92	[1.90; 3.93]	t(270) = − 5.633	41.22	< 0.001	36.32	< 0.001	9.33	< 0.001
				*p* = 0.816			p < 0.001			*p* < 0.001						
SF-36 Physical functioning	1 vs 2	1.86	[−10.40; 6.68]	t(270) = 0.426	24.31	[−33.04; − 15.58]	t(270) = 5.460	21.78	[−30.78; −12.78]	t(270) = 4.742	77.49	< 0.001	36.9	< 0.001	6.41	< 0.001
				*p* = 0.670			*p* < 0.001			*p* < 0.001						
SF-36 Physical role	1 vs 2	−2.14	[−9.66; 13.94]	t(270) = −0.356	14.57	[−26.64; − 2.51]	t(270) = 2.368	22.40	[−34.85; − 9.96]	t(270) = 3.530	60.68	< 0.001	8.05	< 0.001	3.63	< 0.05
				*p* = 0.722			*p* < 0.05			*p* < 0.001						
SF-36 Bodily pain	1 vs 2	2.00	[−9.39; 5.39]	t(270) = −0.53	18.79	[−26.35; −11.23]	t(270) = 4.872	22.17	[−29.96; −14.37]	t(270) = 5.573	26.23	< 0.001	30.9	< 0.001	8.78	< 0.001
				*p* = 0.596			*p* < 0.001			*p* < 0.001						
SF-36 General health	1 vs 2	−2.57	[−3.20; 8.35]	t(270) = −0.873	11.82	[−17.72; −5.92]	t(270) = 3.925	18.89	[− 24.98; −12.91]	t(270) = 6.084	46.48	< 0.001	16.56	< 0.001	12.03	< 0.001
				*p* = 0.383			*p* < 0.001			*p* < 0.001						
SF-36 Vitality	1 vs 2	−4.43	[−3.19; 12.04]	t(270) = −1.141	14.76	[−22.54; −6.98]	t(270) = 3.718	26.17	[− 34.19; −18.15]	t(270) = 6.393	62.3	< 0.001	22.27	< 0.001	12.02	< 0.001
				*p* = 0.255			*p* < 0.001			*p* < 0.001						
SF-36 Social functioning	1 vs 2	4.64	[−11.85; 2.56]	t(270) = 1.263	10.86	[−18.23; −3.50]	t(270) = 2.891	21.61	[−29.21; −14.02]	t(270) = 5.576	44.1	< 0.001	15.53	< 0.001	7.39	< 0.001
				*p* = 0.208			*p* < 0.01			*p* < 0.001						
SF-36 Emotional role	1 vs 2	1.90	[−13.11; 9.30]	t(270) = 0.33	34.52	[−45.97; −23.10]	t(270) = 5.908	37.06	[−48.87;- 25.25]	t(270) = 6.150	74.54	< 0.001	33.61	< 0.001	12.31	< 0.001
				*p* = 0.739			*p* < 0.001			*p* < 0.001						
SF-36 Mental health	1 vs 2	4.00	[−10.64; 2.64]	t(270) = 1.180	17.99	[−24.78; −11.20]	t(270) = 5.192	17.93	[−24.93; −10.93]	t(270) = 5.019	81.15	< 0.001	35.92	< 0.001	6.56	< 0.001
				*p* = 0.239			*p* < 0.001			*p* < 0.001						

Note: *M* Mean; *SE* Standard error; *pre Rehab* Before rehabilitation; *DASH* Disability Arm Shoulder Hand questionnaire; *NRS* Numerical Rating Scale; *SF-36* Short Form Health Survey; Group 1 = Experimental (*n* = 35); group 2 = Control (*n* = 35)

### Primary outcome

**Fig. 2:**
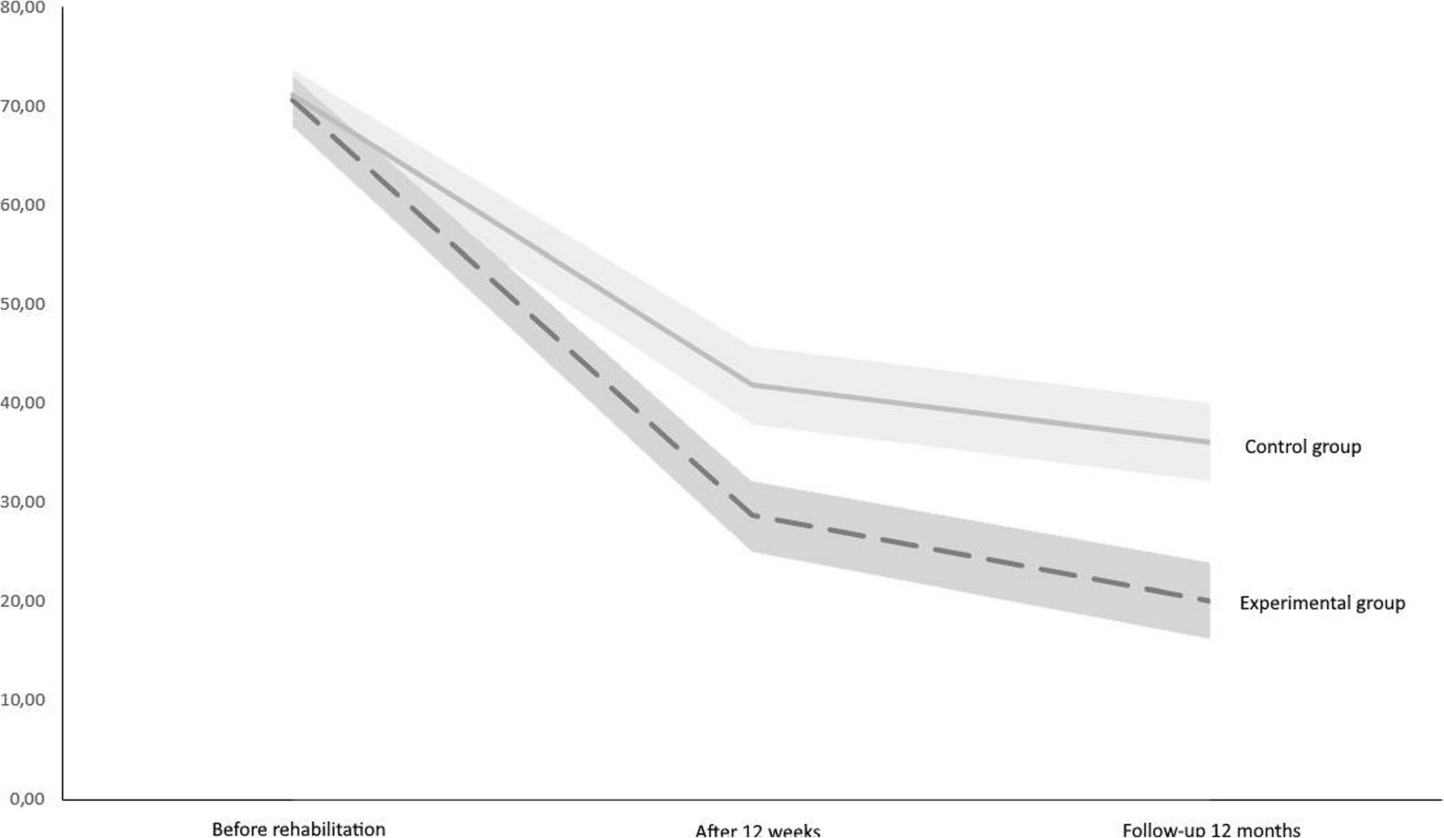
DASH changes over time. Line graphs showing changes of the two groups from before rehabilitation and throughout follow-up. Error bars show standard errors

Both groups improved over time in terms of disability (see Fig. 2), changing from 71 to 29% and to 20% in the experimental group and from 71 to 42% and to 36% in the control group at the end of rehabilitation (primary endpoint) and at follow-up (secondary endpoint), respectively. As shown in Table 4, a between-group difference of 13.1 (95% CI 4.69; 21.57) points was found for DASH at the end of rehabilitation (primary endpoint) and this difference further increased to 16.0 points (95% CI 7.26; 24.66) at follow-up (secondary endpoint).

### Secondary outcomes

Significant improvement was found in the experimental group for DASH work, with a between-group change after training of 18.1 (95% CI 7.80; 28.57) at the end of rehabilitation (primary endpoint) and of 19.8 (95% CI 9.03; 30.45) at follow-up (secondary endpoint). The significant interaction effect indicates the experimental group achieved significantly larger improvement over time with respect to the control group.

Significant improvement was found in the experimental group for NRS, with a between-group change after training of 2.8 at the end of rehabilitation (primary endpoint) and of 2.9 at follow-up (secondary endpoint). The experimental group achieved significantly larger improvement over time with respect to the control group as showed by the significant interaction effect.

As for SF-36, significant improvements were shown in the experimental group, with between-group changes ranging from 10.9 up to 34.5 points considering all domains at the end of rehabilitation (primary endpoint); these differences further improved at follow-up (secondary endpoint), ranging from 17.9 up to 37.0. Again, the significant interaction effect indicates the experimental group achieved significantly larger improvements over time with respect to the control group.

The experimental group had a median GPE significantly lower (1; higher effectiveness of treatment) than the control group (3) (U = 5.879, *p* < 0.001) at the end of treatment (primary endpoint).

The analysis showed there were no significant differences in DASH work levels by the type of work and the origin of the injury.

### Adherence to interventions and adverse effects

Our interventions revealed high rates of adherence to interventions during treatment at the Rehabilitation Unit (95%) and at home (90%). Adverse effects consisted of pain worsening (experimental group: *n* = 5, control group: *n* = 7), easily managed by symptomatic drugs and brief resting periods.

## Discussion

The rehabilitation program that involved task-oriented exercises and occupational therapy (experimental group) was superior to general physiotherapy (control group) in improving disability, pain, and the quality of life of working patients with surgically treated PHFs. The between-group difference was clinically meaningful for our primary outcome (DASH > 10) [14]. Improvements lasted for at least 12 months after the end of the intervention.

Patients need fast recovery and optimization of post-surgery rehabilitation despite evidence on exercises characteristics is still missing [6, 22]. Both approaches analyzed in this study allowed a rapid mobilization of the upper limb, adding evidence to early physical interventions, and being in contrast to a previous study which found no difference between 2 week versus 3 weeks immobilization [23]. Also, task-oriented exercises showed better estimates on disability. Probably, as these kinds of exercises improve functional outcomes and allow for a faster return to normal activities, they generate positive attitudes towards active training and recovery of physical performance in patients. In addition, this study specifically investigated job-related techniques, preferences and amount of time spent during common performances, as per DASH-work subscale. Interestingly, we found that task-oriented exercises were more effective when carried out under conditions alike the ones encountered during job activities. Satisfactory levels in disability were maintained until the end of follow-up, with higher estimates in favour of the experimental group, confirming the possibility of implementing these exercises during common work activities the patients returned at.

In both groups pain perception had reduced by the end of treatment and follow-up periods. This reflects the positive combined effect of surgery and exercises [24].

This study showed that the treatment group had correlated benefits on both physical and mental dimensions of the SF-36. These results confirm the significant role of this novel rehabilitation program in enhancing mental features such as vitality and emotional role. The results of the comparison between experimental and control groups showed the superiority of the intervention. This result can be interpreted in the light of the patients’ perception of the intervention as a better solution to the problems experienced after PHF, also in relation to the work activities previously performed.

Patients suffering from PHFs tend to be frail [6]. Therefore, we chose an outpatient setting involving different rehabilitation professionals such as physiatrists, physiotherapists, and occupational therapists, the first mainly dedicated to physical examination and the others to the delivering of exercises and ergonomic principles, respectively. Each professional was dedicated in particular to the rehabilitation needs of patients and their working environment. Furthermore, the proposed experimental program is to be considered low cost, as the Italian health system has quantified the cost of the complete rehabilitation program in about 350 euros\per person. This program is focused on segmental exercises and although it is slightly more expensive than traditional Italian approaches, the positive effects on pain, disability, and QoL highlighted in this study suggest how it might also play a crucial role in preventing additional costs related to the use of drugs or requests for assistance and limitations in work. Our randomized controlled trial had adequate sample size and we were able to understand the distinct effects in both groups. Also, it was based on (1) concealed randomization, (2) blinded data collection, and (3) effective masking of assessors and analysts. The small number of dropouts from either group, also suggests that the patients were determined and motivated to adhere to all treatment phases. Probably, support offered by both staff and patients’ relatives was a central in creating a controlled and protected context.

The population enrolled displayed clinical and socio-demographic characteristics similar to patients with PHFs and undergoing conservative or surgical treatment in Italy and the United Kingdom [25, 26]. However, outcome measures slightly differed in the above studies and, therefore, a full comparison cannot be stated, therefore this fact partially limits the generalizability of our results. The DASH levels by the type of work and the origin of the injury showed that the effectiveness of the treatment is independent of the category of work performed and the origin of the injury (work-related or non-work-related).

Despite encouraging results, this study has some limitations. First, we did not investigate their relationships with physical measures or tests so we used only self-reported measures. Second, issues regarding treatment-time differences between the treatment groups due to the occupational intervention may be raised. Third, treatment expectations were not discussed with the patients and during the recruitment this confounding factor was only partially limited by telling the patients that the efficacy of both treatments was unknown, and that both methods might benefit the patient by increasing their function. Fourth, despite the focus being on workers, return to work was not included as an outcome measure; researchers are recommended to include it in future studies. Finally, physiotherapists were not blinded to treatment and, therefore, a performance bias could not be excluded [27].

## Conclusion

The findings propose an intervention based on task-oriented exercises as a more effective and long-lasting way to regain disability, pain and quality of life after surgically-treated PHFs rather than a program that involves general exercises where arm deficits are trained as separate components. Therefore, general physiotherapy should be progressively avoided in clinical practice, in favour of exercises which promote functional outcomes in order to guarantee earlier returns to pre-fracture physical levels. Future research should verify the usefulness of task-oriented exercises in younger and older patients after PHFs.

## Data Availability

Anonymized source data can be obtained from the corresponding author on reasonable request.

## Abbreviations

PHF: Proximal humeral fracture
DASH: Disability arm shoulder hand questionnaire
ADL: Activities of daily living
QoL: Quality of life
NRS: Numerical rating scale
SF-36: Short-form health survey-36
GPE: Global perceived effect

## References

[1] Kim SH, Szabo RM, Marder RA. Epidemiology of humerus fractures in the United States: nationwide emergency department sample, 2008. Arthritis Care Res 2012;64(3):407–414, https://doi.org/10.1002/acr.21563. 10.1002/acr.21563 22162357

[2] Launonen AP, Lepola V, Saranko A, Flinkkilä T, Laitinen M, Mattila VM. Epidemiology of proximal humerus fractures. Arch Osteoporos 2015;10:2, https://doi.org/10.1007/s11657-015-0209-4. 10.1007/s11657-015-0209-4 25675881

[3] Dauwe J, Walters G, Holzer LA, Vanhaecht K, Nijs S. Failure after proximal humeral fracture osteosynthesis: a one year analysis of hospital-related healthcare cost. Int Orthop 2020;44:1217–1221. https://doi.org/10.1007/s00264-020-04577-y. 10.1007/s00264-020-04577-y PMC7260263 32342142

[4] Leigh JP. Economic burden of occupational injury and illness in the United States. Milbank Q 2011;89:728–772, https://doi.org/10.1111/j.1468-0009.2011.00648.x. 10.1111/j.1468-0009.2011.00648.x PMC3250639 22188353

[5] Häussler B, Gothe H, Göl D, Glaeske G, Pientka L, Felsenberg D. Epidemiology, treatment and costs of osteoporosis in Germany—the BoneEVA study. Osteoporos Int 2007;18(1):77–84, https://doi.org/10.1007/s00198-006-0206-y. 10.1007/s00198-006-0206-y 17048064

[6] Jawa A, Burnikel D. Treatment of proximal humeral fractures. JBJS Rev 2016;4:1–9, https://doi.org/10.2106/JBJS.RVW.O.00003. 10.2106/JBJS.RVW.O.00003 27490005

[7] Handoll HH, Brorson S. Interventions for treating proximal humeral fractures in adults. Cochrane Database Syst Rev 2015;(11):CD000434, https://doi.org/10.1002/14651858.CD000434.pub4. 10.1002/14651858.CD000434.pub4 26560014

[8] Roberson TA et al. Nonoperative management versus reverse shoulder arthroplasty for treatment of 3- and 4-part proximal humeral fractures in older adults. J Shoulder Elb Surg 2017;26, 1017–1022, https://doi.org/10.1016/j.jse.2016.10.013. 10.1016/j.jse.2016.10.013 28139385

[9] Schumaier A, Grawe B. Proximal Humerus fractures: evaluation and Management in the Elderly Patient. Geriatri Orthop Surg Rehabil 2018;9:1–11*.* https://doi.org/10.1177/2151458517750516. 10.1177/2151458517750516 PMC5788098 29399372

[10] Beks RB et al. Operative versus nonoperative treatment of proximal humeral fractures: a systematic review, meta-analysis, and comparison of observational studies and randomized controlled trials J Shoulder Elb Surg 2018;27, 1526–1534, https://doi.org/10.1016/j.jse.2018.03.009. 10.1016/j.jse.2018.03.009 29735376

[11] Schulz KF, Altman DG, Moher D; CONSORT group. CONSORT 2010 statement: updated guidelines for reporting parallel group randomised trials. J Clin Epidemiol 2010;63(8):834–840, https://doi.org/10.1136/bmj.c332. 10.1016/j.jclinepi.2010.02.005 20346629

[12] Baierlein SA (2011). Frakturklassifikationen.

[13] Hudak PL, Amadio PC, Bombardier C (1996). Development of an upper extremity outcome measure: the DASH (disabilities of the arm, shoulder and hand) [corrected]. The Upper Extremity Collaborative Group (UECG). Am J Ind Med.

[14] Padua R, Padua L, Ceccarelli E, Romanini E, Zanoli G, Amadio PC, Campi A. Italian version of the disability of the arm, shoulder and hand (DASH) questionnaire. Cross-cultural adaptation and validation. J Hand Surg Br 2003;28(2):179–186, https://doi.org/10.1016/s0266-7681(02)00303-0. 10.1016/s0266-7681(02)00303-0 12631494

[15] Huskinson EC. Measurement of pain. Lancet. 1974;2:1127–1131, https://doi.org/10.1016/s0140-6736(74)90884-8. 10.1016/s0140-6736(74)90884-8 4139420

[16] Apolone G, Mosconi P. The Italian SF-36 survey: translation, validation and norming. J Clin Epidemiol 1998;51(11):1025–1036, https://doi.org/10.1016/s0895-4356(98)00094-8. 10.1016/s0895-4356(98)00094-8 9817120

[17] Apolone G, Mosconi P, Ware J (2000). Questionario sullo stato di salute SF-36. Manuale d'uso e guida all'interpretazione dei risultati. [SF-36 quality of life questionnaire. User’s Manual and guide to the interpretation of results].

[18] Kamper SJ, Ostelo RWJG, Knol DL, Maher CG, de Vet HCW, Hancock MJ. Global Perceived Effect scales provided reliable assessments of health transition in people with musculoskeletal disorders, but ratings are strongly influenced by current status. J Clin Epidemiol. 2010;63(7):760–6.e1, https://doi.org/10.1016/j.jclinepi.2009.09.009. 10.1016/j.jclinepi.2009.09.009 20056385

[19] van de Water AT, Shields N, Davidson M, Evans M, Taylor NF. Reliability and validity of shoulder function outcome measures in people with a proximal humeral fracture. Disabil Rehabil. 2014;36(13):1072–9, https://doi.org/10.3109/09638288.2013.829529. 10.3109/09638288.2013.829529 24001265

[20] Fielding S, Fayers P, Ramsay CR. Analysing randomised controlled trials with missing data: choice of approach affects conclusions. Contemp Clin Trials 2012 May;33(3):461–469, https://doi.org/10.1016/j.cct.2011.12.002. 10.1016/j.cct.2011.12.002 22265924

[21] Siddiqui O, Hung HMJ, O’Neill R. MMRM vs. LOCF: a comprehensive comparison based on simulation study and 25 NDA datasets. J Biopharm Stat 2009;19(2):227–246, https://doi.org/10.1080/10543400802609797. 10.1080/10543400802609797 19212876

[22] Haldoll HH. Developing, delivering and documenting rehabilitation in a multi-Centre randomised controlled surgical trial. Bone Joint Res 2014;3:335–340, https://doi.org/10.1302/2046-3758.312.2000364. 10.1302/2046-3758.312.2000364 PMC4286698 25519445

[23] Wirbel R, Knorr V, Saur B, Duhr B, Mutschler W (1999). Minimally invasive fixation of displaced proximal humeral fractures. Orthop Traumatol.

[24] Dunlop DD, Song J, Semanik PA, et al. Physical activity levels and functional performance in the osteoarthritis initiative: a graded relationship. Arthritis Rheum 2011;63(1):127–136, https://doi.org/10.1002/art.27760. 10.1002/art.27760 PMC3010474 20862681

[25] Carbone, S., Razzano, C., Albino, P., Mezzoprete, R. Immediate intensive mobilization compared with immediate conventional mobilization for the impacted osteoporotic conservatively treated proximal humeral fracture: a randomized controlled trial. Musculoskelet Surg 2017;101:137–143, https://doi.org/10.1007/s12306-017-0483-y. 10.1007/s12306-017-0483-y 28770512

[26] Rangan A, Handoll H, Brealey S, Jefferson L, Keding A, Martin BC, Goodchild L, Chuang LH, Hewitt C, Torgerson D; PROFHER trial collaborators. Surgical vs nonsurgical treatment of adults with displaced fractures of the proximal humerus: the PROFHER randomized clinical trial. JAMA 2015;313(10):1037–1047, https://doi.org/10.1001/jama.2015.1629. 10.1001/jama.2015.1629 25756440

[27] Boutron I, Moher D, Altman DG, Et al; CONSORT group. Extending the CONSORT statement to randomized trials of nonpharmacologic treatment: explanation and elaboration. Ann Intern Med 2008; 148(4): 295–309, https://doi.org/10.7326/0003-4819-148-4-200802190-00008. 10.7326/0003-4819-148-4-200802190-00008 18283207

